# Altered pH gradient at the plasma membrane of osteosarcoma cells is a key mechanism of drug resistance

**DOI:** 10.18632/oncotarget.11503

**Published:** 2016-08-22

**Authors:** Sofia Avnet, Silvia Lemma, Margherita Cortini, Paola Pellegrini, Francesca Perut, Nicoletta Zini, Katsuyuki Kusuzaki, Tokuhiro Chano, Giulia Grisendi, Massimo Dominici, Angelo De Milito, Nicola Baldini

**Affiliations:** ^1^ Orthopaedic Pathophysiology and Regenerative Medicine Unit, Istituto Ortopedico Rizzoli, Bologna, Italy; ^2^ Department of Oncology-Pathology, Cancer Center Karolinska, Karolinska Institute, Stockholm, Sweden; ^3^ CNR - National Research Council of Italy, Institute of Molecular Genetics, Bologna, Italy; ^4^ Laboratory of Musculoskeletal Cell Biology, Istituto Ortopedico Rizzoli, Bologna, Italy; ^5^ Musculoskeletal Oncology Unit, Takai Hospital, Nara, Japan; ^6^ Department of Clinical Laboratory Medicine, Shiga University of Medical Science, Otsu, Shiga, Japan; ^7^ Department of Medical and Surgical Sciences for Children and Adults, University-hospital of Modena e Reggio Emilia, Modena, Italy; ^8^ Department of Biomedical and Neuromotor Sciences, University of Bologna, Bologna, Italy

**Keywords:** osteosarcoma, doxorubicin, drug resistance, plasma membrane pH gradient, tumor microenvironment

## Abstract

Current therapy of osteosarcoma (OS), the most common primary bone malignancy, is based on a combination of surgery and chemotherapy. Multidrug resistance mediated by P-glycoprotein (P-gp) overexpression has been previously associated with treatment failure and progression of OS, although other mechanisms may also play a role. We considered the typical acidic extracellular pH (pHe) of sarcomas, and found that doxorubicin (DXR) cytotoxicity is reduced in P-gp negative OS cells cultured at pHe 6.5 compared to standard 7.4. Short-time (24–48 hours) exposure to low pHe significantly increased the number and acidity of lysosomes, and the combination of DXR with omeprazole, a proton pump inhibitor targeting lysosomal acidity, significantly enhanced DXR cytotoxicity. In OS xenografts, the combination treatment of DXR and omeprazole significantly reduced tumor volume and body weight loss. The impaired toxicity of DXR at low pHe was not associated with increased autophagy or lysosomal acidification, but rather, as shown by SNARF staining, with a reversal of the pH gradient at the plasma membrane (ΔpH_cm_), eventually leading to a reduced DXR intracellular accumulation. Finally, the reversal of ΔpH_cm_ in OS cells promoted resistance not only to DXR, but also to cisplatin and methotrexate, and, to a lesser extent, to vincristine. Altogether, our findings show that, in OS cells, short-term acidosis induces resistance to different chemotherapeutic drugs by a reversal of ΔpH_cm_, suggesting that buffer therapies or regimens including proton pump inhibitors in combination to low concentrations of conventional anticancer agents may offer novel solutions to overcome drug resistance.

## INTRODUCTION

Osteosarcoma (OS) is the most common primary malignancy of bone [1]. Since the introduction of multiagent chemotherapy to surgical removal of the primary lesion, the 5-year survival of OS patients has significantly improved, although 30–40% of localized OS and over 70% of metastatic OS do not respond to therapy and develop pulmonary metastases [2]. The mechanism of resistance to chemotherapy has been extensively investigated in OS [3–5]. Most studies have focused on pathways modulating cancer cell sensitivity, such as the transmembrane ATP-dependent efflux pump P-glycoprotein (P-gp) [3–6], Her-2/ERBB2 [7], the Bcl-2 family proteins [8], and, more recently, miRNAs [9, 10]. On this respect, however, the role of tumor microenvironment (TME), a key player for tumor cell survival at the primary lesion and the receptive soil for cancer cell seeding at distant sites for the (pre)-metastatic niche [11, 12], has been neglected so far. In many cancers [13–17], including OS [18, 19], a number of TME-dependent factors may play a key role in chemoresistance, including the modification of the extracellular matrix, the recruitment of stromal cells, and hypoxia. The hypoxic TME of OS, an osteogenic malignancy usually arising in bone, may be particularly relevant for tumor behavior. In fact, bone is *per se* a hypoxic tissue with an oxygen tension between < 1% in hypoxic region and < 6% in proximity of sinusoidal cavities [20], and it is well known that hypoxia controls a number of relevant bone tissue-specific activities, including angiogenesis, recruitment of stem precursors, proliferation, and differentiation of committed osteogenic elements [21]. Tumor cells cope with hypoxia by switching from aerobic respiration to glycolysis, in turn producing lactic acid and causing extracellular acidosis [22]. In several malignancies, the increased reliance on glycolysis to produce energy occurs even in the presence of sufficient oxygen supply [23, 24]. Indeed, the extracellular pH (pHe) of different tumor types, including sarcomas, ranges from 6.4 to 7.3, whereas the pHe of normal tissues is in the range of 7.2–7.5 [25]. Locally and acutely, intratumoral pH varies from one area to another, showing a trend of decrease that in the long term (chronically) results into an average persistent intratumoral acidosis. Indeed, pH can locally and rapidly change due to a short-lived phenomenon, like to apoptosis of a small group of cells, to temporary hypoxia due to the disruption of small vessels, or to temporary high glycolytic activity [26, 27]. As a result, in the tumor TME, acidosis is both chronic and acute, with different grading. We have recently demonstrated in sarcomas that a low pHe is linked to malignant behavior [28, 29]. In other cancers, acidity has also been associated with drug resistance [30–32].

In this study, we studied the role of pH regulation on drug resistance of OS. For this purpose, in wild type OS cells we investigated doxorubicin (DXR) cytotoxicity and intracellular accumulation under acidic conditions, the role of lysosomal acidification and autophagy on drug resistance, and the effects of lysosomal pH modification both *in vitro* and *in vivo*. We investigated on the effect of rapid changes in pH on drug effectiveness. Our results show that, besides P-gp overexpression, extracellular acidity is another major player of resistance to DXR, suggesting the use of pH modifiers as novel therapeutic tools to increase the efficacy and reduce toxicity of conventional anticancer agents.

## RESULTS

### The acidic TME of OS is associated with resistance to DXR

**Figure 1:**
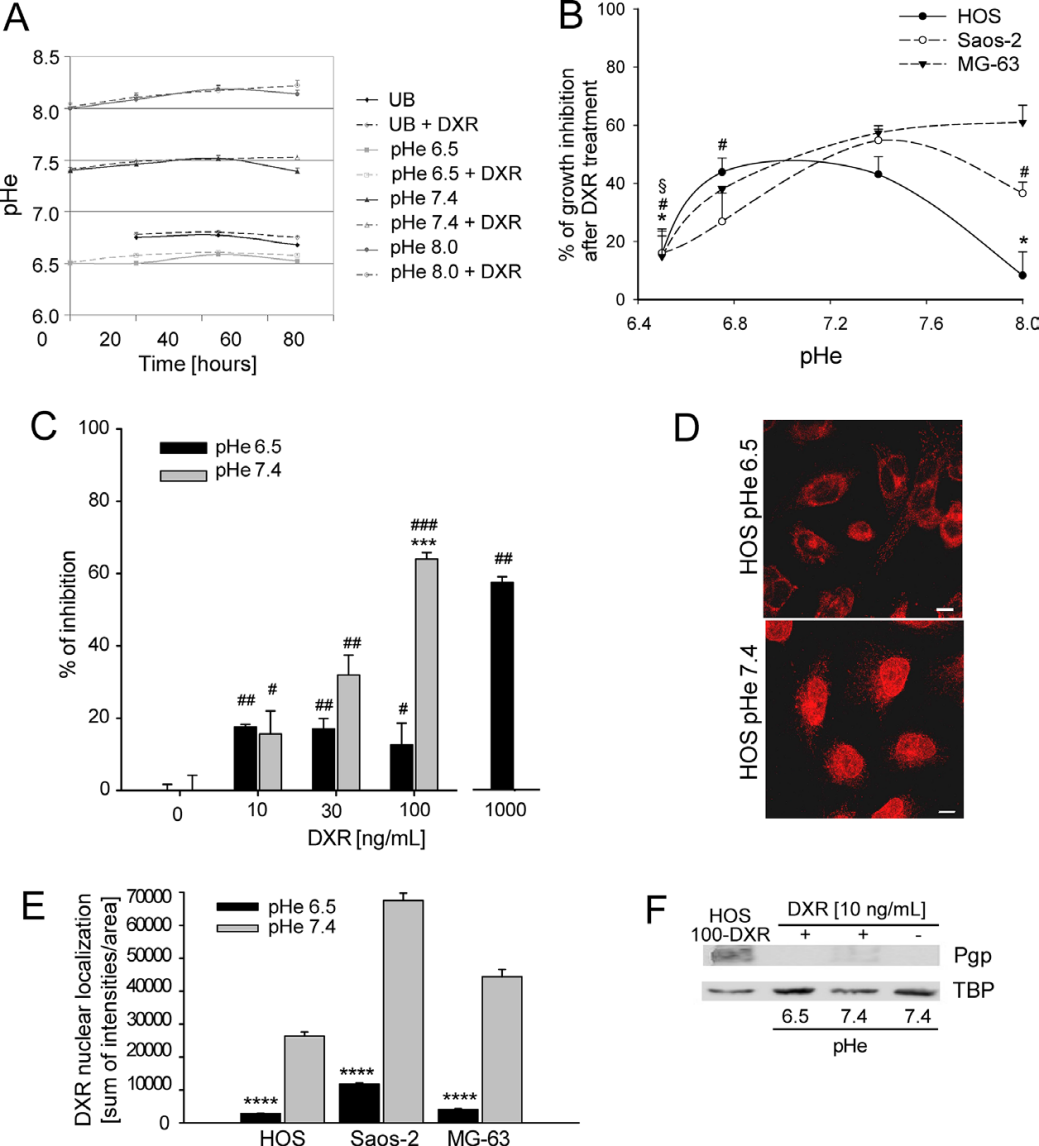
Chemoresistance induced by low pHe (**A**) Measurement of pH of medium buffered at different pH and unbuffered (UB), over the culture period; (**B**) percentage of growth inhibition of osteosarcoma (OS) P-glycoprotein (P-gp) negative cells cultured at different pH and treated with Doxorubicin (DXR) (15 ng/mL) by direct cell counting. Growth inhibition was obtained in respect to the untreated condition at the respective pH (*p < 0.05 vs pH 7.4 for HOS; ^#^*p* < 0.05 vs pH 7.4 for Saos-2; ^§^*p* < 0.05 vs pH 7.4 for MG-63); (**C**) Percentage of growth inhibition of HOS P-gp negative cells at different concentrations of DXR under acidic conditions by viability indirect assay (***p < 0.001 vs pH 7.4 at the same concentration; ^#^*p* < 0.05, ^##^*p* < 0.01, and ^###^*p* < 0.001 vs not treated at the same respective pH); (**D**) DXR nuclear compartmentalization in HOS P-gp negative cells maintained at different pH for 48 h, observation at the confocal microscope (Scale bars = 5 μm). (**E**) Fluorescence intensity of DXR in the nuclei of OS P-gp negative cells treated with 10 mg/mL DXR (****p < 0.0001); (**F**) western blotting analysis of Pgp expression in HOS P-gp negative cells at different pHe and treated or not treated with DXR for 30 days. HOS 100-DXR P-gp positive cells were used as positive control. HOS 100-DXR cells were obtained after continuous exposure to DXR for several months at pHe 7.4.

As proof-of-principle that our *in vitro* models were representative of the acidic TME of OS, we verified if the preselected pHe (culture medium at pH 6.5–7.4–8.0) at the beginning of the culture, with or w/o DXR, was maintained over the incubation period. We checked the pH of medium at different time points for all the OS cell lines included in this study and it was very similar between the different cell lines. After 72 h, the pHe was slightly decreased, possibly due to the high number of sub-confluent cells. However, the specific pHe values were stable over the culture period (Figure 1A, representative values only for HOS cells). As expected, due to its cationic nature, DXR induced a trend of a slight increase in pHe at all conditions. In unbuffered medium, HOS cells secreted an amount of protons that, combined with the 5% of atmosphere CO_2_, induced a pHe of around 6.8 (at 48 h: 6.76 ± 0.09, *n* = 6, Figure 1A).

We then sought to investigate whether the reduced cytotoxicity of DXR under an acidic TME was present in OS cells, even if they do not express P-gp, as previously observed in other cancers [31–33]. Indeed, following DXR exposure, the percentage of growth inhibition of OS P-gp negative cells calculated by direct cell counting significantly correlated with the pH values (Figure 1B). By indirect viability test, we also found that, at pHe 6.5, the concentration of DXR needed to obtain 60% of growth inhibition in the same cells was 10-fold higher than at pHe 7.4 (Figure 1C). The same type of indirect test was also used to calculate EC50 of DXR at different pH. EC50 at pH 6.5 was around 2,000–fold higher with respect to pH 7.4 in P-gp negative cells, whereas the expression of P-gp in HOS 100-DXR cells that we used as positive control for chemoresistance (P-gp positive cells) induced an increase of the EC50 value of only 500-fold in respect to sensitive (P-gp negative) cells (Table 1). To exclude the possibility that the reduced DXR cytotoxicity of OS cells cultured at low pHe was due to cell death promoted by acidity, we used Annexin-V test. We found no difference in apoptosis in untreated HOS P-gp negative cells cultured at acidic pH in respect to neutral pH (Supplementary Figure S1, CTR), whereas DXR-treated P-gp negative cells showed a significantly higher induction of apoptosis under neutral pH in respect to cells cultured at pH 6.5 (Supplementary Figure S1, DXR 50 ng/mL). Interestingly, culturing sensitive cells (P-gp negative cells) at acidic pHe for 48 h was sufficient to completely impair DXR nuclear accumulation (Figure 1D and 1E). Resistance to DXR mediated by acidic pHe in sensitive cells was not associated with the induction of P-gp expression by acidosis since HOS P-gp negative cells were still negative for the expression of P-gp protein also when cultured at pH 6.5 for 1 month (Figure 1F, HOS 100-DXR P-gp positive cells are the positive control).

**Table 1:** IC50 of DXR for growth inhibition

	DXR [ng/mL]	*R*^2^
**pHe 6.5**	2303.40	0.9952
**pHe 7.4**	1.37	0.9978
**HOS 100-DXR**	505.49	0.9599

### Low pHe enhances lysosomal activity but not the autophagic flux

The doubling rate of OS sensitive (P-gp negative) cells cultured under acidity was 1.5–2.6 times slower than under standard conditions (Table 2), and this reduced doubling rate is possibly partially causing the impaired cytotoxicity of DXR.

**Table 2:** Doubling time of OS cell lines at different pHe

	pHe 6.5	pHi 7.4	*p*
HOS	33.0 ± 0.6 (*n* = 4)	14.3 ± 0.7 (*n* = 4)	0.0209
MG-63	72.3 ± 13.8 (*n* = 5)	28.2 ± 2.7 (*n* = 4)	0.0143
Saos-2	38.3 ± 2.0 (*n* = 5)	25.1 ± 4.4 (*n* = 4)	0.0275

**Figure 2:**
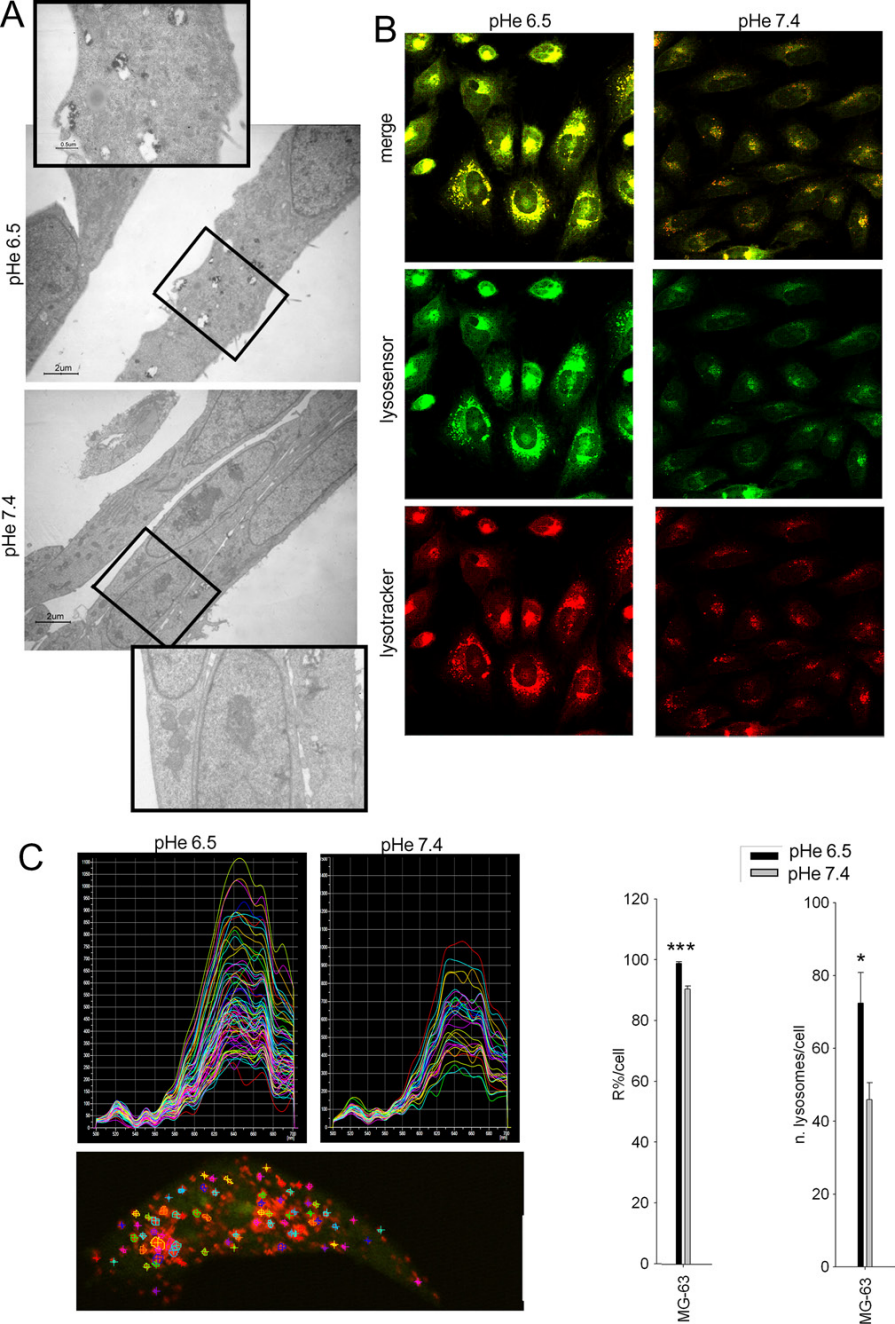
Lysosomes in cells under acidic conditions are more acidic and increased in number OS cells were maintained for 48 h at different pHe before analyses. (**A**) trasmission electron microscopy of HOS cells (in the rectangles, a detail of the original image is enlarged); (**B**) lysosensor (green) and lysotracker (red) staining of HOS cells; (**C**) spectrum analysis of Acridine Orange (AO) emission in vesicle-like structure in HOS cells. AO is a probe for measuring pH gradients across membranes with particular specification to intravesicular acidification, with a trend of localization and subsequent accumulation and polymer formation (emission at 655 nm) in acidic compartment, and of monomer localization at higher pH (emission at 530 nm). Upper panels: representative graphs of the intensity of spectra of all the lysosomes within a cell at the two different pHe; lower panel: representative image of intracytoplasmic area examined; right panel: quantification of the lysosomal acidity by spectra analysis [R% = 100* I_655_/ (I_655_ + I_530_) where I_655_ and I_530_ are the green (520–540 nm) and the red (645–665 nm) integrated emission intensities, ***p < 0.0001 vs pHe 7.4)], and number of acidic vesicles within a cell (*p < 0.05 vs pHe 7.4).

We next reasoned to assess the role of lysosomal acidification on the acid-induced chemoresistance. The number of lysosomes of HOS cells was increased and lysosomes appeared also more activated when cultured in acidic medium, as shown by the presence of a higher number of residual bodies detected by electron microscopy, with respect to the neutral condition (Figure 2A). To determine lysosomal pH, cells were co-stained with Lysotracker and Lysosensor fluorescent dyes that showed selective accumulation in the lysosomal compartment. Cells cultured under acidic conditions had a striking increase in the number of acidic vesicles and thus a lower vesicular pH (Figure 2B). To further extend these findings, we performed quantitative measurement of the intensity of the fluorescence emission spectra of Acridine Orange (AO) uptaken by single cells at different wavelengths. AO is a probe for measuring pH gradients across membranes with particular specification to intravesicular acidification. Indeed, MG-63 cells cultured in acidic medium showed a shift from the green (alkaline) to the red (acidic) emission wavelength, indicating acidification of lysosomes (Figure 2C). Furthermore, the number of lysosomes per single cell was significantly increased (Figure 2C). Lysosomal activity is strongly associated with mTOR activity, and the recovery of the autophagic flux is associated with the early inhibition of mTOR phosphorylation, previously reported to occur during acidic stress [34, 35]. Therefore, we investigated whether OS sensitive cells under acidic conditions showed an altered transcriptome related to the mTOR. This, however, was not the case: in fact, after 24 h we did not observe any significant variation of the transcripts related to the mTOR pathway (Supplementary Figure S2).

**Figure 3:**
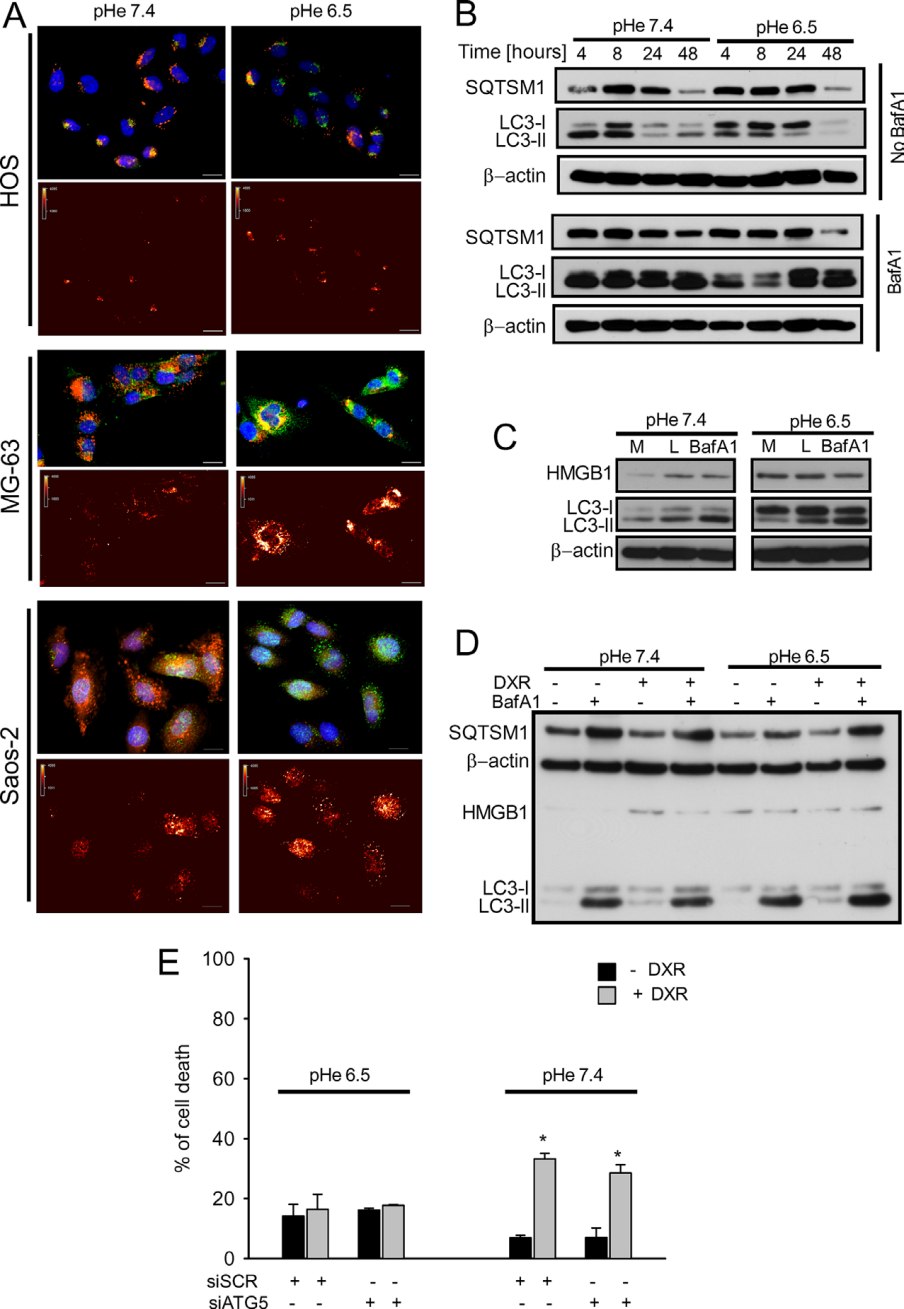
Autophagic flux was unaffected in OS cells cultured under acidic conditions (**A**) Upper panel: LC3-B immunostaining (green), with LysoTracker staining (red), and nuclei staining (blue) under different pH (scale bar 10 μm). Lower panel: false color for LC3-B staining showed in the upper panel; (**B**) analysis of autophagic flux by western blotting of LC3B and SQTSM1 expression of HOS cells cultured at pH 7.4 and 6.5 at different pH and at different time-points (4-8-24-48- hrs) in presence of absence of BafA1treatment (50 nM) for 2 h before collection; (**C**) western blot analysis of proteins related to the autophagic flux HMGB1 and LC3-II in HOS cells cultured at pH 7.4 and 6.5, in presence of BafA1 (50 nM) for 2 hrs before collection, or of the lysosomal inhibitors (L, Pepstain A/E64d) treatments at 10 μg/mL, or with complete medium (M); (**D**) western blot analysis HMGB1, LC3-II, and SQTSM1 in HOS cells treated for 24 h with DXR, in presence or absence of BafA1 at different pH (7.4 and 6.5); (**E**) evaluation of the effect of the silencing of autophagic protein ATG5 on HOS cell death, under acidic conditions in HOS cells (*p < 0.05 vs not treated cells at the respective pH).

Staining for the autophagic markers LC3 (green) and lysotracker (red) showed the presence of LC3-positive lysosomes (Figure 3A). In order to understand how low pH modulates autophagy in OS cells, we performed kinetics analysis of the autophagic flux in HOS cells after 48 h. During autophagy, the cytosolic form of LC3 (LC3-I) is lipidated to the autophagosome-associated form (LC3-II) which is then degraded after fusion with lysosomes. Measurement of the autophagic flux is determined by the accumulation of LC3-II protein in the presence of the lysosomal inhibitor bafilomycin A1 (BafA1) and expressed as the ratio of LC3-II signal intensity in cells with and without BafA1 and normalized for the actin signal [36]. Despite a temporary but minor downregulation of the autophagic flux at 4-8 h, cells were fully competent for autophagy at 24 and 48 h, as indicated by the similar accumulation of LC3-II in the presence of BafA1 in the two pH conditions (Figure 3B and Supplementary Figure S3). The ability of HOS cells to maintain a high self-degradative process after exposure for 24 h at pH 6.5 was also confirmed by using other lysosomal inhibitors (Figure 3C). Since upregulation of the autophagy modulator HMGB1 was reported to mediate DXR resistance in OS [37], we evaluated the effects of acidosis on intracellular HMGB1 levels in HOS cells. Both DXR and acidity *per se* induced the expression of HMGB1 in HOS cells (Figure 3C and 3D and Supplementary Figure S4). Despite this, treatment with DXR did not affect the autophagic flux in HOS cells in neither of the pH considered (Figure 3D and Supplementary Figure S5). In order to study whether the maintenance of autophagy in acidic conditions was associated with sensitivity to DXR in HOS cells, we performed transient siRNA-mediated knock-down of Atg5, a gene involved in autophagosome maturation. After efficient knockdown of Atg5 (Supplementary Figure S6), cells were exposed to medium at pH 7.4 or pH 6.5 and treated or not treated with DXR. Analysis of cell death showed that inhibition of autophagy does not affect the sensitivity to DXR under either pHe (Figure 3E). In addition, cells cultured at acidic pH maintained DXR resistance independently of autophagy. In conclusion, these findings show that modulation of autophagy during acidity is not involved in DXR resistance of OS cells.

### Targeting lysosomal activity enhances the DXR effectiveness

**Figure 4:**
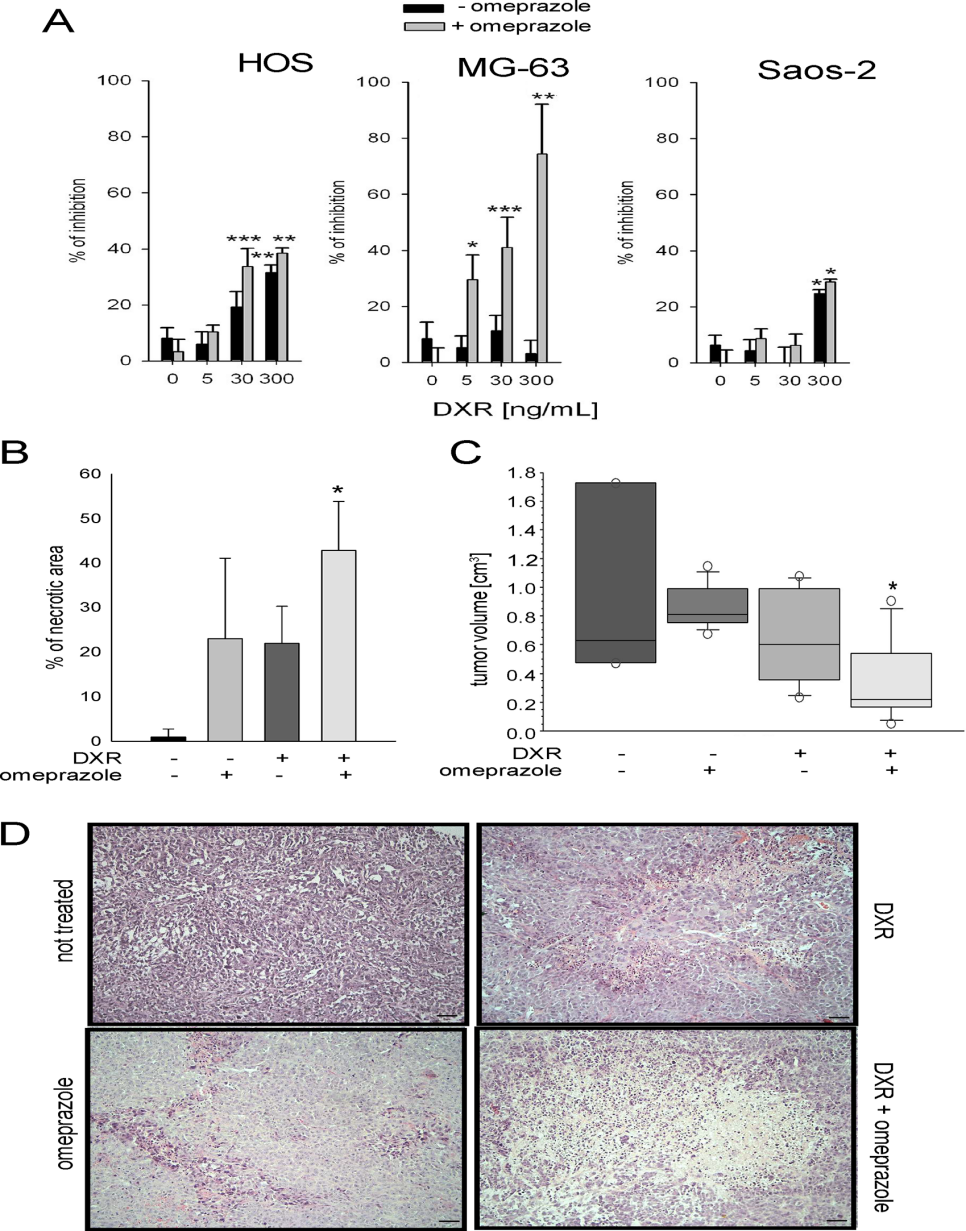
Lysosomal targeting enhanced DXR cytotoxicity (**A**) Cells were treated with DXR for 48 h under acidity following pretreatment of 2 h with (+ OME) or without (- OME). The percentage of inhibition was obtained by an indirect viability assay (*p < 0.05, **p < 0.01, and ***p < 0.001 vs not DXR treated cells, at the respective +OME or – OME condition); (**B**) percentage of necrotic area in a mouse model of OS treated with DXR in combination or not with OME (*p < 0.05 vs non treated group); (**C**) Tumor volume in the same xenografts (*p < 0.05 vs non treated group); (**D**) ematoxylin & eosin staining of xenograft sections, representative images (scale bar = 100 micrometers).

To confirm that the autophagy-independent lysosomal activity is involved in DXR response after short-term acidosis, we used omperazole (OME), a proton pump inhibitor that targets lysosomal acidification by blocking vacuolar-ATPase (V-ATPase) activity in sarcoma cells [29, 38]. Treatment with OME enhanced growth inhibition promoted by DXR under acidity. Especially in HOS and MG-63 sensitive cells, DXR treatment showed a significant effect at lower doses, when used in combination with OME, with respect to the single treatment (Figure 4A).

Similar results were obtained *in vivo* in a xenograft model of OS. When both intraperitoneally administered, combined treatment with OME (10 mg/kg) enhanced the cytotoxic effect of DXR (1.5 mg/kg), resulting in a significantly lower tumor volume and a higher necrotic index (Figure 4B–4D). Interestingly, mice treated with the combination of OME and DXR showed a statistically significant reduced loss of body weight compared to mice treated with DXR alone (DXR treated group: 20.1 ± 0.6 gr; OME + DXR treated group: 22.3 ± 0.6 gr; *p < 0.05), possibly suggesting that OME reduced DXR toxicity.

### Altering the pH gradient at the plasma membrane is a key mechanism of drug resistance

**Figure 5:**
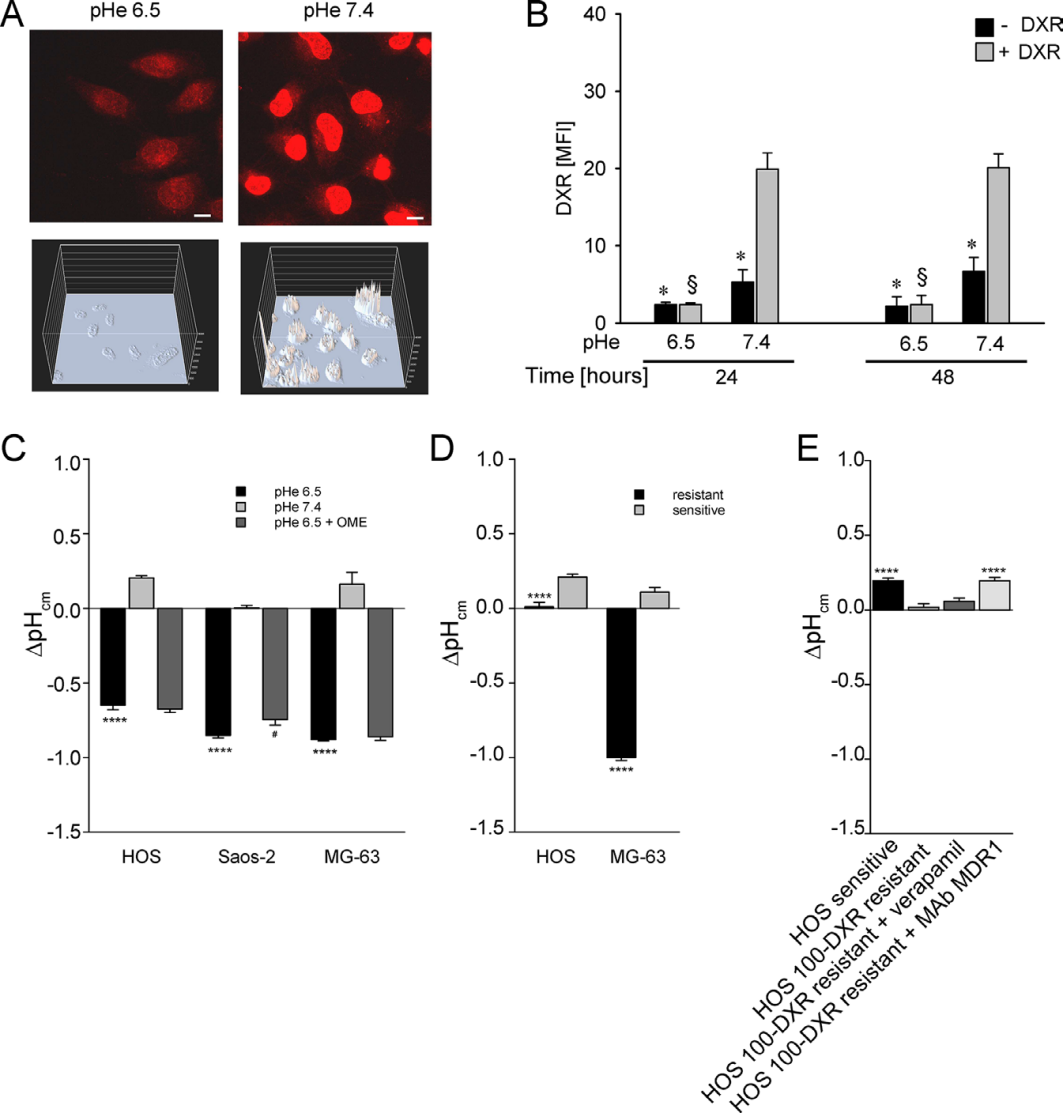
Total DXR concentration in HOS cells under acidity is reduced due to an increased pH gradient (ΔpHcm) at the plasma membrane (**A**) 3D intensity embossed and confocal acquisition of DXR fluorescence in the DXR intracellular uptake assay by NIS Element software (Nikon) in HOS cells cultured under different pH, representative image (scale bar = 10 μm); (**B**) cytofluorimetric quantification of DXR uptake in HOS cells cultured under different pHe (*p < 0.05 not treated vs the DXR treated, at the same pHe; ^§^ *p* < 0.05 DXR treated at pHe 6.5 vs the DXR treated at pH 7.4). MFI, Mean Fluorescence Intensity. (**C**) ΔpH_cm_ plasma membrane gradient by SNARF staining of live cells cultured under acidic conditions, treated or not treated with OME, and measured by spectral confocal microscopy (****p < 0.0001 vs pHe 7.4, ^#^*p <* 0.05 vs pHe 6.5, *n* = 12). The uncharged SNARF-1-AM can passively diffuse across the plasma membrane to cytosol, where it hydrolyzed by cellular esterases to free SNARF-1. It is this form that is fluorescent, and is consequently the intracellular pH probe. Intracellular pH measurement and pH calibration using single excitation-dual emission fluorescence ratios were performed as described in the method paragraph; (**D**) ΔpH_cm_ plasma membrane gradient by SNARF staining of live resistant cells cultured at pHe 7.4 and that express P-gp protein (****p < 0.0001 vs sensitive cultured at pHe 7.4, *n* = 12); (**E**) ΔpH_cm_ plasma membrane gradient by SNARF staining of live HOS resistant cells (HOS 100-DXR), cultured at pHe 7.4, and treated or not treated with verapamil or with a monoclonal blocking antibody against MDR-1 (****p < 0.0001 vs resistant cells).

**Figure 6:**
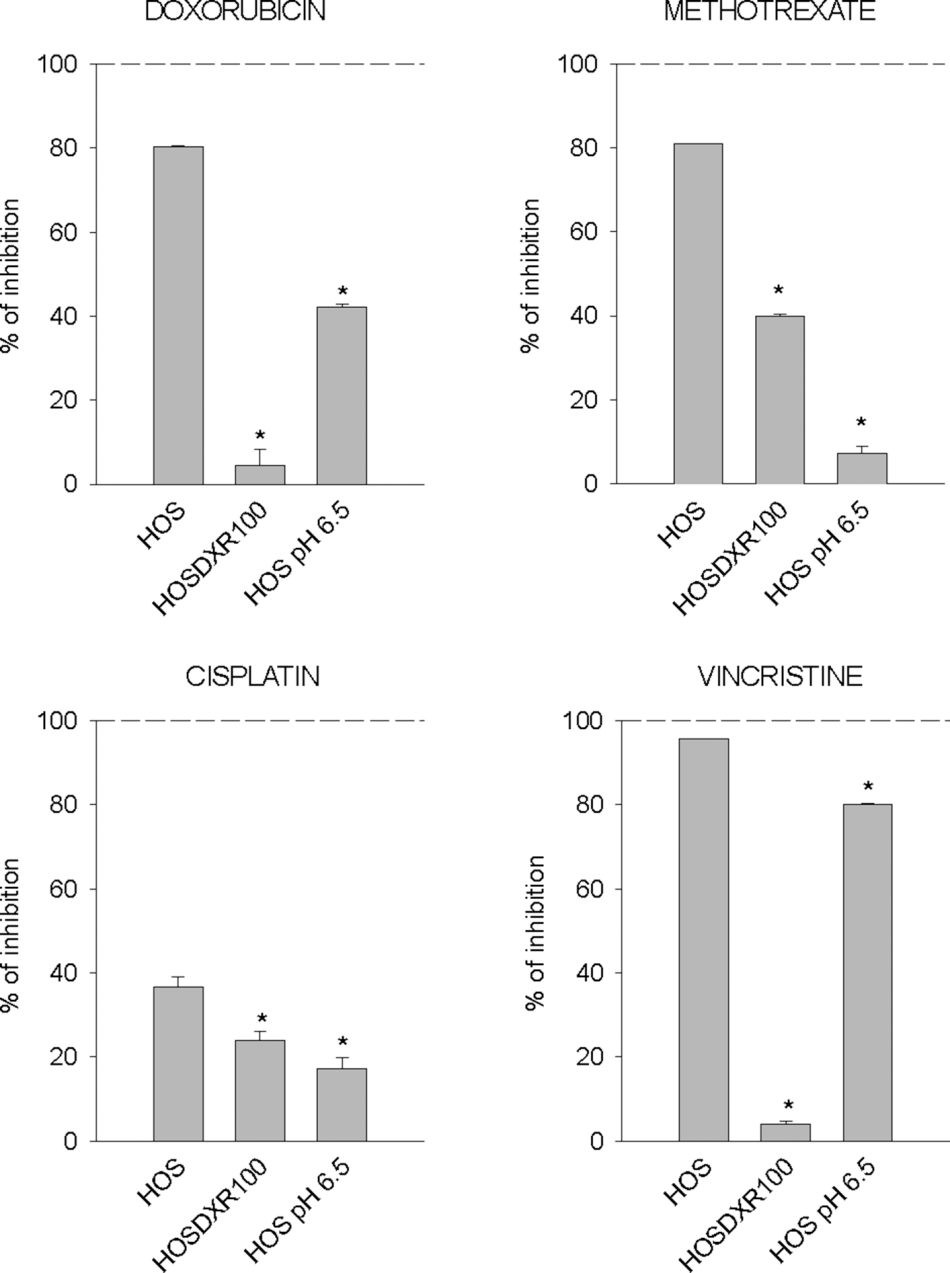
Chemoresistant phenotypes of OS cells cultured at different pHe and treated with different drugs Percentage of inhibition of cell viability of HOS cells cultured at pH 7.4, of P-gp expressing HOS cells (HOS 100-DXR), and of HOS cells cultured at pH 6.5 treated with different drugs, as measured by acid phosphatase assay after 72 h (*p < 0.05 vs HOS cells cultured at pH 7.4 and treated with the respective drug).

We then sought to investigate the total amount of DXR that effectively enters into the OS sensitive (P-gp negative) cells cultured under acidic conditions through image analysis (Figure 5A) and flow cytometry (Figure 5B). A low fluorescent signal of the drug was detected within the cells at acidic pH in respect to neutral conditions both at 24 and 48 h (Figure 5B). This finding suggests that the major obstacle for DXR effectiveness under short-term acidosis is its entrance into the cell through the plasma membrane. This is possibly due either to a phenomenon known as the partition theory, or through a change of membrane stiffness, both related to a reversal of the ΔpH_cm_ [33]. To extend this observation, we used SNARF staining in living cells and observed a significant alteration of ΔpH_cm_ that would result in an enhancement of intracellular positive charge with respect to the extracellular space (Figure 5C). The alteration of ΔpH_cm_ showed a trend of reduction in MG-63 cells and a significant reduction in Saos-2 sensitive (P-gp negative) cells when cells were treated with OME (Figure 5C). Interestingly, we found that the same pattern of an altered ΔpH_cm_ was also present in P-gp expressing resistant OS cells cultured in a standard medium at pH 7.4, with respect to the sensitive P-gp negative parental cells also cultured in standard medium at pH 7.4 (Figure 5D). The use of the MDR modifier verapamil produced a decrease of this effect, whereas the incubation with a MDR-1 neutralizing antibody significantly impaired this effect, completely restoring the original phenotype (Figure 5E). These results suggest that chemoresistance mediated also by P-gp activity is partially due to pHi regulation. Finally, we found that drug resistance promoted by short-term acidosis is not specific only to DXR, since the treatment with methotrexate, vincristine, and at a lower extent with cisplatin was significantly impaired by low pH (Figure 6). As a control we used P-gp expressing cells in which a very high resistance to DXR and vincristine, and only a low resistance to cisplatin and methotrexate were present. Notably, drug resistance conferred by short-term acidosis was dramatically higher for methotrexate, and less evident with vincristine. In the first case, it is likely that BCRP cancer resistance proteins that efflux methotrexate out of the cells and that are expressed by OS cells [39], transport more efficiently the drug at low pH [40]. Regarding vincristine, the mild effect of pH 6.5 on resistance is possibly due to the very acidic pKa of the drug (between 3.5 and 4.5) that is thus less subject to the reversal of ΔpH_cm_.

## DISCUSSION

Since the time of discovery of MDR mediated by P-gp expression [41], the concept of chemoresistance has been extensively revised and expanded into two major types. Biochemical resistance is mediated by the upregulation of drug efflux or metabolic pathways, such as MDR-1 or BCRP. Moreover, physiological resistance may also occur as a consequence of poor perfusion, hypoxia, and/or acidity [42].

In this study, we mimicked the acidic conditions of extracellular space that are commonly detected in OS as a result of the significant anomalies in energy metabolism by using *in vitro* cell culture medium buffered at pH 6.5. Under these conditions, we observed that the growth inhibition of OS sensitive (P-gp negative) cells induced by DXR was significantly impaired. In fact, at pH 6.5, a 10-fold increase of DXR concentration was necessary to achieve the same level of growth inhibition detected at pH 7.4, and the amount of drug that accumulated into the nucleus, the main target site of DXR activity, was significantly reduced.

Therefore, the effect of pH on DXR resistance did not rely on P-gp expression, and might be attributed to different mechanisms. We observed a reduced replication rate at pHe 6.5, possibly due to a block in cell cycle [43], and this might interfere with the effectiveness of an antiproliferative agent, like DXR, blocking type II topoisomerase activity [44]. Another possible explanation concerns lysosomal acidification. Acidic lysosomes can sequester weakly basic molecules, like DXR, to an extent that is directly related to the level of acidity [45, 46]. The compartmentalization into the lysosomes might derive from the transfer of DXR from the cytosol, or from a direct endocytotic process [47]. Compared to standard conditions, in OS cells cultured for a few hours in acidic medium, we found a higher number of larger lysosomes and, as already demonstrated for breast cancer cells [46], the lysosomal compartment was also more acidic.

Closely associated to lysosomal acidification, autophagy is an adaptation mechanism that cancer cells adopt when exposed to an adverse microenvironmental conditions, such as acidity [35, 48] and anticancer agents, including DXR [37]. After 24 h exposure to acidity, however, we did not observe changes in the expression of autophagy-related genes. Such a short endpoint was selected to parallel the rapid nuclear uptake of DXR. Although treatment with DXR or with acidic pHe increased HMGB1 expression, the inhibition of autophagy via knock-down of Atg5 did not alter the sensitivity of OS cells to DXR at any pHe condition, further suggesting that autophagy-mediated DXR resistance is not a general phenomenon in OS cells.

We further investigated the role of lysosomes in OS chemoresistance under short-term acidic conditions, and independently of autophagy. We have previously shown that treatment with OME, a gastric H+/K+-ATPase inhibitor that targets also the lysosomal proton pump V-ATPase [49], alkalinizes lysosomal pH in sarcoma cells [29, 38]. Here, we reasoned to test the effects of OME on DXR sensitivity, both *in vitro* and *in vivo*. After pretreatment with OME, the growth inhibition of DXR was significantly increased. The combination of OME with DXR induced a growth rate reduction at a 10-fold lower concentration of DXR. In the xenograft model of OS, when combined with OME, treatment with DXR produced a significant reduction of tumor volume and an increase of necrosis. Most importantly, these effects were obtained with a low concentration (1.5 mg/kg) of DXR with respect to the dosage commonly used in OS xenografts (2.5 mg/kg) [50]. DXR as a single treatment at the same concentration did not produce any effect. Such concentration was calculated as 1/6 of the minimum dose used for a therapeutic regimen in human patients (60–90 mg/m^2^, corresponding to 9–14 mg/kg in a child of 24 kg, with a height of 120 cm) [1]. The combined treatment also had a positive impact on the systemic toxicity associated with DXR treatment. In fact, mouse body weight was less affected with respect to DXR alone both at 1.5 mg/kg and 2.5 mg/kg (data not shown).

In summary, the reduced toxicity of DXR in OS sensitive (P-gp negative) cells under low pHe was independent of autophagy but apparently associated with an increased lysosomal acidification. Notably, this type of resistance develops within hours, in contrast to the expression of P-gp that occurs, according to the canonical model of MDR, after months of continuous exposure to DXR. To verify that resistance to DXR under short-term exposure to acidity is actually due to drug accumulation into lysosomes, we observed the intracellular fluorescence of DXR after 5–10 min of incubation. Surprisingly, DXR did not accumulate into the cell, a minor portion being compartmentalized into lysosomes. The high pH gradient generated between the acidic extracellular compartment and the alkaline cytosolic space is sufficient to affect the permeability to weak cationic drugs at the plasma membrane [51]. This occurs because uncharged, organic free bases are more permeable than their protonated charged counterparts and establish equal concentrations on both sides of the membrane. We therefore measured the difference between the extracellular and the cytosolic pH (pHe-pHi, ΔpH_cm_) at different pHe. Although the pHi was mildly acidified by the extracellular milieu, the net result was a strong alteration of ΔpH_cm_ with a reversal of net charges. Notably, in Saos-2 cells, pre-treatment with OME slightly reduced this trend. Only a few reports have linked pHi regulation also to drug resistance mediated by P-gp expression. MDR cells have been reported to have a more alkaline pHi [32, 52], and the magnitude of plasma membrane electrical potential decreases along with the increase of expression of P-gp [53]. According to this hypothesis, P-gp-mediated effects depend also on an altered regulation of ion transporters and signal transduction pathways (i.e., pH and membrane potential, key features necessary for the correct setting the biophysical parameters of the cells) [54]. In this context, P-gp has been suggested to work as a chloride channel, although it is yet unclear whether it might physically form a channel-like structure or rather regulate nearby anion transporters involved in the maintenance of pHi [53, 55, 56]. Here, we confirmed that P-gp expression in P-gp overexpressing OS cells obtained by continuous exposure to DXR [5] induces an alteration of ΔpH_cm_, that is partially reverted by verapamil. Moreover, we further verified that the anti P-gp monoclonal antibody MRK16, known to revert the MDR phenotype [57] completely abolished the ΔpH_cm_ decrease observed under acidity. Therefore, according to our data, a change in ΔpH_cm_ is necessary to induce chemoresistance, either by short-term acidosis or by the long term drug exposure leading to P-gp expression. On this regard, the reversed ΔpH_cm_ has been recently shown to interfere with the passage of drugs across the lipid bilayer of the cells, by altering the fluidity, the relative lipid density, and the surface tension of each of the leaflets within the membrane [33]. Short-term acidosis induced chemoresistance not only to DXR, but also to methotrexate, vincristine and, at a lesser extent, to cisplatin, other drugs commonly included in the standard therapeutic protocol for OS.

### Concluding remarks

**Figure 7:**
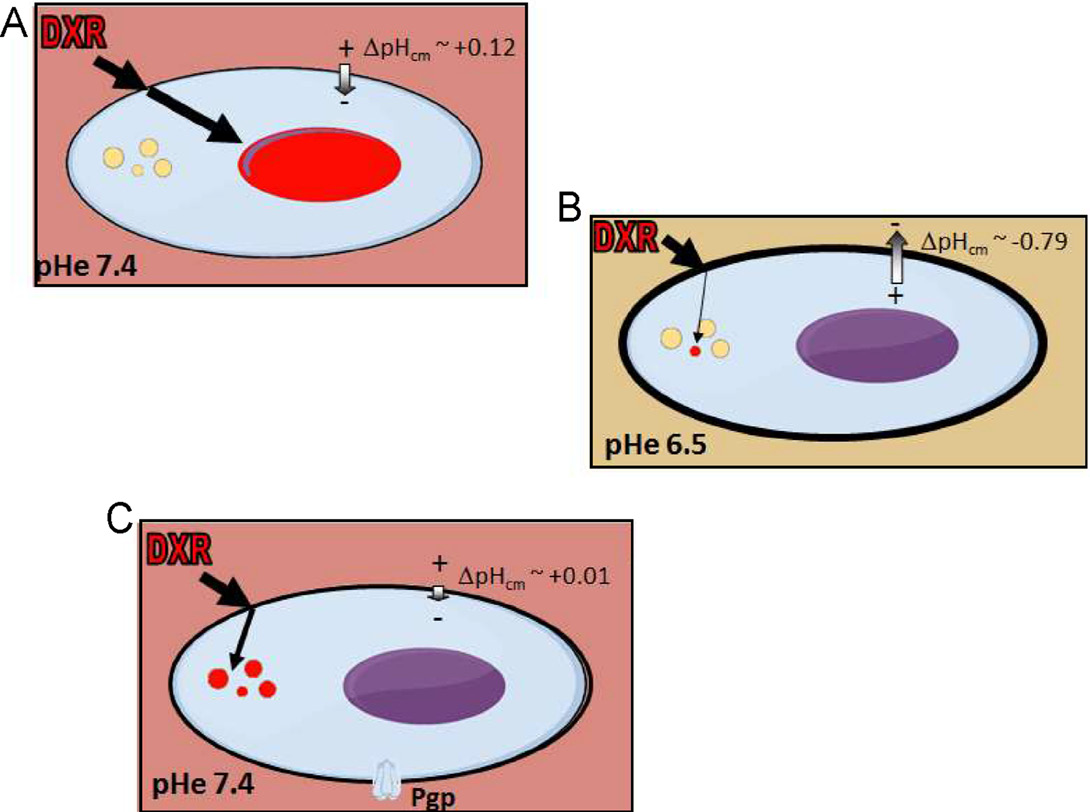
ΔpH_cm_ at the plasma membrane is an important player in OS chemoresistance (**A**) When OS cells growth in a microenvironment with pHe 7.4 and with an unaffected system to maintain the pHi, DXR accumulates into the nucleus; (**B**) when OS cells growth in a microenvironment with pHe around 6.5, the ΔpH_cm_ is reverted and DXR does enter into the cell. Only a very small fraction of DXR reaches the cytoplasm, and is immediately sequestered into lysosomes that, under this condition, are even more acidic; (**C**) when OS cells growth in a microenvironment with pHe 7.4 and express Pgp, the system to maintain the pHi is altered, membrane potential is reduced, and lysosomes are more acidic. As a result, although DXR can reach the cytoplasm, it is immediately sequestered into lysosomes.

Altogether, our data provide evidence that the chemoresistant phenotype of OS is dependent on an inverted or altered ΔpH_cm_. This might rapidly result from the acidification of the TME (Figure 7B), or derive from the induction of the expression of P-gp (Figure 7C). In this view, buffer therapies or proton pump inhibitors that block the abnormal ΔpH_cm_ could be used in combination with standard anticancer agents. This hypothesis is supported by the encouraging results obtained in a pilot clinical study of human OS by the addition of esomeprazole to standard chemotherapy regimens [58]. Finally, the consistent evidence that changes of pHe strongly impact on the cytotoxicity of anticancer drugs should be taken into account. We therefore propose that *in vitro* IC50 assays for anticancer agents in sarcomas should always include a condition with an acidic medium in the range of pH 6.5-6.8 to mimic the actual conditions of TME.

## MATERIALS AND METHODS

### Cell lines

HOS, Saos-2, MG-63, and 143B human OS cell lines were purchased from the American Type Culture Collection, cultured in RPMI plus 20 units/mL penicillin, 100 μg/mL streptomycin, and 10% FCS at pH 7.4 (complete medium), and incubated at 37°C in a humidified 5% CO_2_ atmosphere. In assays with different pH, cells were seeded in complete medium, and after 24 h media were changed. New media were set at a specific pH by using different concentrations of sodium bicarbonate needed to preset pH in 5% CO_2_ atmosphere, according to the Henderson-Hasselbach equation. At the end-point of each experiment, the final pH in the supernatant was always measured by a digital pH-meter (pH 301, HANNA Instruments). To obtain HOS 100-DXR cells and MG-63 MDR cells we continuously exposed parental cell lines to stepwise increases in DXR concentration (10-30-100 ng/mL for HOS and 10-30 ng/mL for MG-63, Sigma). Approximately 2–3 weeks (4-5 passages) were required to establish adequate growth at each DXR concentration.

### Growth assays

Cells were seeded in 6-well plates (100,000 cells/well) in complete medium. After 24 h, the medium was changed with new complete medium at different pH, or with complete medium without sodium bicarbonate (unbuffered medium, UB), with or w/o DXR (15 ng/mL). The pH in UB condition, measured at all time points and for each cell line, was always 6.75 ± 0.07. Cell growth was evaluated by direct cell counting using the eritrosin dye (Sigma). Growth inhibition was obtained in respect to the untreated condition at the respective pH, and at a time point just after the specific doubling time at the considered pH (for HOS: 24 h at pH 7.4 and 8, 48 h at pH 6.5 and 6.75; for Saos-2: 48 h at all the pH; for MG-63: 42 h at pH 7.4 and 8, and 72 h at pH 6.5 and 6.75). The experiments were repeated three times, with two replicates for each assay.

To calculate the IC50 values for DXR or to evaluate the growth inhibition of DXR treatment combined with pre-treatment with the proton-pump inhibitor omeprazole (OME), cells were seeded in 96-well plates (4,000 cells/well) in complete medium. After 24 h, the medium was changed with new medium at different pH, with or w/o DXR (0-10-30-100-1000 ng/mL). For the combined treatment, 2 h before the change of media with DXR, cells were washed with PBS and incubated with medium 0.1 % FCS with or w/o OME (210 μM, Sigma). After 72 h since the addition of DXR, the cell number was measured by an acid phosphatase assay, as previously described [59]. Briefly, the culture medium was removed, wells were washed with PBS, and 100 μl of buffer containing 0.1 M sodium acetate (pH 5.0), 0.1% Triton X-100, and 5 mM p-nitrophenil phosphate (Sigma) was added. After 3 h at 37°C, the reaction was stopped with the addition of 10 μl of a 1 N NaOH, and color development was assayed at 405 nm using a microplate reader (Tecan Infinite F200pro). The experiment was repeated twice in quadruplicate.

Similarly, to evaluate the percentage of inhibition of viability, cells were seeded in complete medium, and after additional 24 h, the medium was replaced with the respective conditions added with DXR 100 ng/mL, or Cysplatin 250 ng/mL, or Methotrexate 10 ng/mL, or Vincristine 100 ng/mL. After 72 h the cell number was measured by an acid phosphatase assay as described above. The experiment was repeated twice in quadruplicate.

### DXR uptake

According to the previously described method [60], cells were seeded at low density in a glass-bottom Petri dish (Inco), and incubated for 48 h in complete medium at different pH. Then, the medium was changed at the respective pH with DXR 10 μg/mL for 5 or 15 min. Cells were washed with PBS and maintained with drug-free complete medium at the respective pH. For quantitative determination of intranuclear DXR concentration, the laser (Exc wavelength 457.9 nm) was focused for 1 sec with a pinhole of 54 μm to the nucleus of cells to obtain fluorescence emission spectra. The drug concentration in the nuclei of living cells were obtained from the specific spectra contribution of DXR (Emi 560–590 nm). The analysis was performed using a TiE spectral confocal laser microscope (A1R, Nikon) equipped with a DS-QIMc-U2 12 bit camera. We measured the fluorescence of 25 nuclei (within the nuclear perimeter) of 5 different random optical fields. DXR nuclear uptake was expressed as the mean of the sum of intensities divided by the nuclear area. The same experimental conditions and settings for confocal microscopy were also used for the acquisition of 3D intensity embossed images.

### Western blotting

HOS cells were seeded in complete medium. After 24 h the medium was changed with new medium at different pH with or w/o DXR (10–100 mg/mL) for 1 month to determine the level of P-gp expression. The medium was changed every 3–4 d, and confluent cells were trypsinized. Cells were treated in ice with 400 of lysis buffer [50 mM HEPES (pH 7.5), 150 mM NaCl, 1.5 mm MgCl2, 1 mM EGTA, 10% glycerol, 1% Triton X-100, 1% PMSF, 0.2 mM Na orthovanadate and 1% aprotinin, all from Sigma]. Protein concentration was determined with the Bradford protein assay (Bio-Rad). Total protein was separated on 6% gradient SDS-PAGE gel and transferred into nitrocellulose membranes. Blots were blocked with 5% nonfat dry milk in TBST and probed with the monoclonal P-gp (clone D-11), or TATA binding protein (TBP) rabbit antibodies (Santa Cruz Biotechnology). The secondary antibody was an anti-rabbit or anti-mouse IgG conjugated to horseradish peroxidase that was visualized by enhanced chemiluminescence detection reagents (Pierce). For the analysis of autophagy flux in HOS cells, PVDF membranes (GE Healthcare) were used and incubated with the following antibodies: rabbit polyclonal LC3B (Cell Signaling Technology), monoclonal β-actin (ACTB, clone AC-15) (Sigma), rabbit polyclonal ATG5 (Cell Signaling Technology), monoclonal SQSMT1 (clone 3/P62 LCK LIGAND, BD Transduction laboratories), and rabbit DNA binding protein high mobility group box 1 (HMGB1) (Cell Signaling Technology) antibodies.

### Ultrastructural analysis

HOS cells cultured under acidic (pH 6.5) or standard (pH 7.4) conditions were washed in PBS and fixed with 2.5 % glutaraldehyde in 0.1 M cacodylate buffer pH 7.4 (Sigma) for 30 min at R°T, scraped, and pelletted at 1,300 g for 20 min. Pellets were further fixed for 2 h. After washing in 0.1 M cacodylate buffer, samples were post-fixed with 1% osmium tetroxide in cacodylate buffer (Sigma) for 1 h at 4°C, dehydrated, and embedded in Epon resin. Ultrathin sections were stained with tannic acid, uranyl acetate and lead citrate, and observed with a Zeiss EM 109 transmission electron microscope. Image were captured using a Nikon digital camera (Dmx 1200F) and the ACT-1 software.

### Lysosomal pH assays

Cells were cultured in complete medium at different pH for 48 h and then analyzed. *Lysosensor staining:* labeling and tracking of acidic organelles in living cells was performed by incubation with 1 μM LysoSensor Green DND-189 and 50 nM LysoTracker Red DND-99 (Molecular Probes, Life Technologies) in medium at the respective pH. After 30 min at 37°C, cells were washed with fresh medium and observed by confocal microscopy (Nikon Eclipse E600).

*Confocal spectral analysis:* the emission spectra of the pH-sensitive dye AO was used to measure pH variations in acidic organelles, as previously described [61]. MG-63 cells were incubated with AO (0.5 μg/mL, Sigma) in medium at the respective pH for 15 min. After washing, x, y emission spectra from confocal sections of single living cells were recorded using a confocal laser microspectrofluorimeter (Nikon, TI) equipped with an argon-ion laser. Cells were focused with a ×40, 1.3 NA objective (S Fluor, Nikon), excited at 457 nm and the resulting fluorescence emission in the 500–700 nm range was collected. For intracellular measurements of AO emission the pinhole size was fixed to a diameter of 54 μm. To characterize the profile of AO emission spectra, the red band contribution (R%) within the whole emission spectrum was calculated as follows: R%=100* I_655_/ (I_655_ + I_530_) where I_655_ and I_530_ are the green (520–540 nm) and the red (645-665 nm) integrated emission intensities, respectively. The R% was calculated for all the lysosomes within a single cell, and the average R% of lysosomes of single cells was considered. Average of the total number of all the lysosomes per single cell that were detectable by confocal microscopy analysis and AO staining was calculated out of 20 cells.

### RNA extraction and RNA sequence analysis

Total RNA was extracted using guanidinium thiocyanate-phenol-chloroform from cell cultured under acidic (pH 6.5) or standard conditions (pH 7.4) for 24 h, and quantified by Bioanalyzer (Agilent, Santa Clara, CA) following the manufacturer's instructions. RIN (RNA Integrity Number) and A260/A280 ratio of the prepared total RNA were all 10, and over 1.8, respectively. The library of template molecules for high throughput DNA sequencing was converted from the total RNA using TruSeq RNA Sample Prep Kit v2 (Illumina) following the manufacturer's protocol. The library was quantified with Bioanalyzer (Agilent) following the manufacturer's instruction. Library (7 pM) was subjected to cluster amplification to cluster generation on a Single Read Flow Cell v4 with a cluster generation instrument (Illumina). Sequencing was performed on a Genome Analyzer GAIIx for 76 cycles using Cycle Sequencing v4 regents (Illumina). Image analysis and base calling were performed using Off-Line Basecaller Software 1.6 (Illumina). Reads were aligned using ELAND v2 of CASAVA Software 1.7 with the sequence data sets. Human genome build 19 (hg19) were downloaded from University of California, Santa Cruz genome browser (http://genome.ucsc.edu/) as the analytic reference. Transcript coverage for every gene locus was calculated from the total number passing filter reads that mapped, by ELAND-RNA, to exons. These analyses were performed using default parameters. The advanced analysis for quantification with Quantile normalization algorithm was performed using Avadis NGS software (version1.5, Strand Scientific Intelligence Inc.). Filtering was performed using default parameters. All new data were deposited in DDBJ/EMBL/GenBank under accession number DRA DRA004087.

### Immunofluorescence

Before fixation, cells were incubated for 24 h at different pH, and then incubated with Lysotracker (0.25 μM) in medium at the respective pH for 30 min at 37°C. Cells were washed with PBS, fixed in 3% paraformaldehyde in PBS containing 300 mM sucrose (Sigma) for 20 min at 22°C. After washing with PBS, permeabilization was performed with 0.1% Triton X-100 for 5 min. Then, cells were incubated with the anti-LC3B antibody (Santa Cruz Biotechnology), and with a solution containing anti-mouse Alexa green 488 nm (Molecular Probes, Life Technologies), and Hoechst 33258 (1.25 μg/mL, Sigma) for the nuclear staining, and observed by confocal microscopy (Nikon TI-E). The staining was repeated two times.

### Analysis of the autophagic flux

HOS cells were seeded at 200,000 cells/plate in 6 cm tissue cultures dishes in standard RPMI medium. After overnight incubation the medium was replaced with medium buffered at pH 7.4 or at pH 6.5 and cells were collected at different time points (4, 8, 24 and 48 h) after exposure to fresh media. For each time point, cells were treated or not with 50 nM bafilomycin A1 (BafA1) for 2 h before collection. In some experiments, the lysosomal inhibitors pepstatin A and E64d were used at 10 mg/ml. Analysis of the effects of DXR where performed by adding the drug (100 nM) for 24 h in the presence or absence of BafA1. The use of BafA1 to block lysosomal acidification was performed in order to analyze the rate of LC3 and SQTM1 degradation, indicative of the autophagic flux [36].

### ATG5 silencing

HOS cells were seeded in 6-wells plates (50,000 cells/well) in medium without antibiotics. The next day, cells were transfected with 25 nM siRNA for ATG5 (Dharmacon RNA Technologies siGENOME SMART pool) and siRNA SCR (scrambled siRNA, control) using DharmaFECT-4 transfection reagent (Dharmacon), following the manufacturer's instructions. The next day, the medium was changed with fresh complete medium buffered at different pH (7.4 and 6.5), with or without DXR (100 ng/mL). After 36 h, cells were collected and analyzed for apoptosis by Annexin V staining. Cells for Western blot analysis were collected at the same time. Four replicates were made for each assay.

### Apoptosis assay

We evaluated apoptotic cell death by using Annexin-V staining (FITC Annexin V Apoptosis Detection Kit, BD Pharmingen) and by collecting green fluorescence in a FACSCalibur flow cytometer (Becton Dickinson) using the Cellquest Pro software. At least 10,000 cells/sample were acquired and analyzed. The experiments were repeated five times, with two replicates for each assay.

### *In vivo* studies

NOD/SCID animals were housed and maintained in a pathogen-free environment. 143B human cells (1 × 10^6^) were subcutaneously injected with the reduced growth factor matrigel (BD, Bioscience) in the flank of 5 weeks old male mice (Charles River Laboratories International). Four days after the inoculation, mice were randomly separated into groups and assigned to pharmacological treatments (for each group *n* = 7). Both OME and DXR were reconstituted in saline solution and injected intraperitoneally (10 mg/kg and 1.5 mg/kg, respectively). Mice received saline solution, OME, DXR or a combination of OME and DXR, the latter being injected 2 h after OME treatment. Weights were taken daily during treatment, and drug concentrations were recalculated to ensure that mice received a constant dose of OME and DXR. Treatments were repeated every 4 days and mice were sacrificed 18 days after tumor inoculation. Tumor size was estimated at every pharmacological treatment with a caliper and the weight was calculated by using the formula: tumor volume [mm^3^] = (length [mm] × width^2^ [mm^2^])/2 [62]. For histological analysis, tumor xenografts were fixed in formalin and embedded in paraffin. 5 μm sections were stained with hematoxylin/eosin. The area of necrosis was quantified by NIS element image analysis software (Nikon). The Animal Ethics Committee of Modena has approved all the experimental protocols.

### DXR intracellular content by flow cytometry quantification

HOS cells were seeded in 6-well plates (50,000 cells/well) in complete medium. The next day, the medium was changed with fresh medium buffered at different pH (7.4 and 6.5), with or w/o DXR (50 ng/mL). After 24 and 48 h, cells were collected and at least 10,000 cells/sample were analyzed by flow citometry. The mean fluorescence intensity (MFI) in the FL2 channel was evaluated by the DXR red fluorescence.

### pH gradient at the plasma membrane

Cytosolic intracellular pH (pHi) of OS cells was measured by using carboxy-SNARF-1 and confocal microspectrofluorometry (Nikon, TI). Cells were seeded into glass Petri dishes and incubated for 48 h in medium at different pH. At the end, cells were additionally incubated in acidic pH (pH 6.5) for HOS, Saos-2, and MG-63 cells or in complete medium for HOS 100-DXR cells, with OME (210 mM) and Verapamil (500 μM), single treatment or combined, or with the MRK16 anti-MDR-1 antibody (10 m/mL). At the time of the analysis, cells were then treated with medium at the respective pH containing 10 μM carboxy-SNARF-1 of the acetoxymethyl ester form (Molecular Probes, Life Technologies) at 37°C with 5% CO_2_ for 30 min. Medium was replaced again with medium at the respective pH and Petri dishes were placed in an incubator (OkoLab), on the stage of the confocal microscope. We used an excitation laser of 514 nm wavelength (Argon) and a S Plan Fluor ELWD 40X lend (Nikon). The resulting fluorescence emissions at 644 nm and at 594 nm wavelengths were collected. Several regions of interest (ROI) were then randomly selected excluding nuclear regions. The emission ratio was calibrated using solutions (110 mM KCl, 25 mM KHCO3, 11 mM glucose, 1 mM MgCl2, 1 mM CaCl2, 10 mM HEPES, Sigma) with varying pH (pH 6.5–8.0) that contained 10 μM nigericin (K^+^/H^+^ ionophore, Sigma). In this case, cells were allowed to adapt for at least 20 min before pHi measurements began. The fluorescence emission ratio (644 nm/594 nm) was calculated and used to estimate cytoplasmic pH from the calibration curve of each cell line. Confocal images were processed using NIS Elements software (Nikon). We quantified 12 different random cells for each condition.

### Statistical analysis

Due to the small number of observations, data were considered as not normally distributed. Values were expressed as means ± SE. Statistical analysis was performed with the StatView 5.0.1 software (SAS Institute Inc., Cary, NC). The nonparametric Mann-Whitney *U* test was used and *p* < 0.05 was considered significant.

## SUPPLEMENTARY MATERIALS FIGURES



## Abbreviations

OS: Osteosarcoma
DXR: doxorubicin
P-gp: P-glycoprotein
TME: tumor microenvironment
HIF-1α: hypoxia-inducible factor-1α
pHe: extracellular pH
MDR: multidrug resistance
pHi: intracellular pH
ΔpHcm: pH gradient at the cytoplasmic membrane
UB: unbuffered medium
OME: omeprazole
TBP: TATA binding protein
HMGB1: DNA binding protein high mobility group box 1
AO: acridine orange
BafA1: bafilomycin A1
