# Supplementary material for: Altered pH gradient at the plasma membrane of osteosarcoma cells is a key mechanism of drug resistance
Source: Oncotarget. 2016 Aug 22;7(39):63408–23. doi: 10.18632/oncotarget.11503 (PMC5325373; doi:10.18632/oncotarget.11503)
Supplement: Supplementary file 1 [file oncotarget-07-63408-s001.pdf]

# Altered pH gradient at the plasma membrane of osteosarcoma cells is a key mechanism of drug resistance

## Supplementary Materials

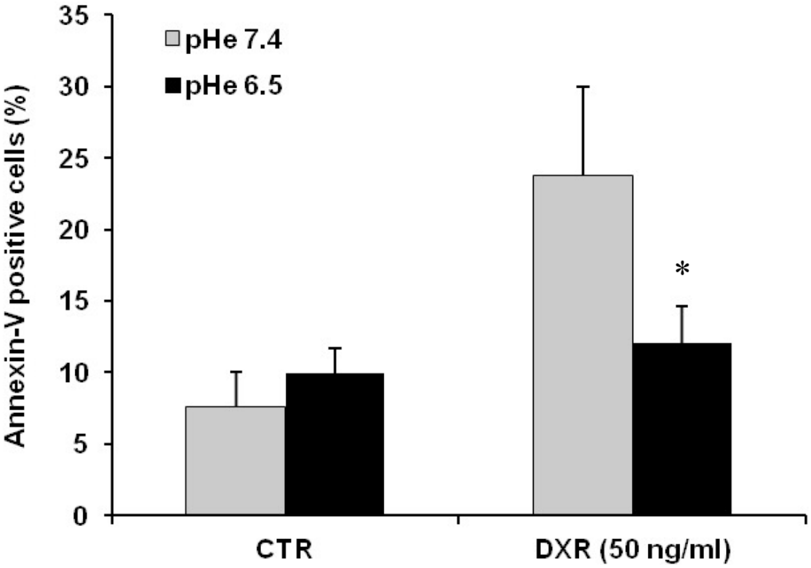


**Supplementary Figure S1: Apoptosis of HOS cells treated with DXR and cultured at different pH.** Annexin-V test (*$p < 0.05$ vs pHe 7.4 at the same DXR treated or not treated conditions).

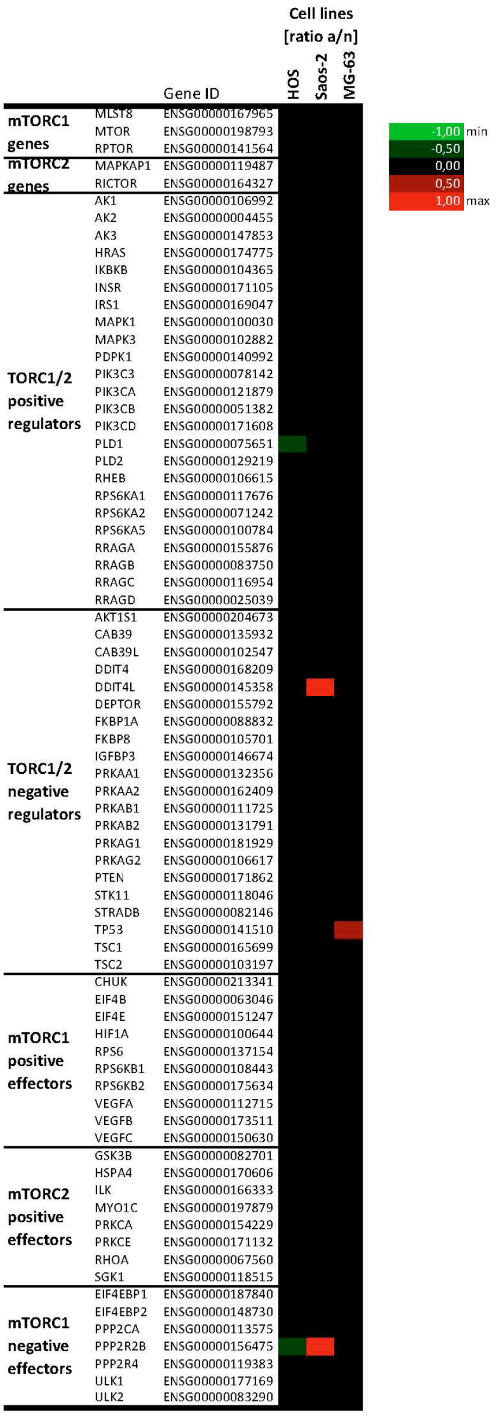


**Supplementary Figure S2: Transcriptome analysis of the autophagic-related genes in OS cells under an acidic TME.** Deep sequencing analysis of 76 autophagy-related genes differentially expressed after 24 h of incubation in acidic medium (pH 6.5) in respect to standard medium (pH 7.4) in HOS, MG-63, and Saos-2 sensitive (P-gp negative) cells. Colors on the heat map indicate the $\log_2$ ratios of expression (representing normalized read counts) in cell cultured in acidic medium in matched cells cultured in standard medium. Red, upregulation; green, downregulation.

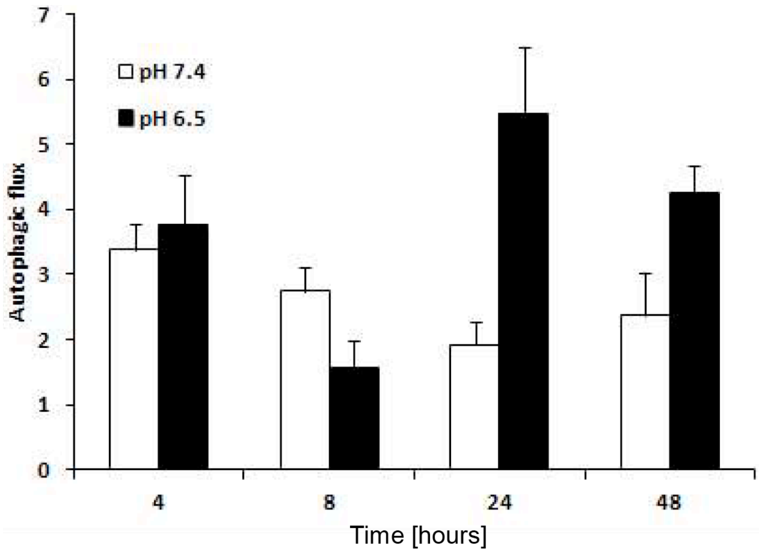


**Supplementary Figure S3: Densitometric quantification of western blot analysis shown in Figure 3B.** HOS cells cultured at pH 7.4 and 6.5 at different pH values and at different time-points (4–8-24–48 h) in presence of absence of BafA1 treatment (50 nM) for 2 h before collection. The autophagic flux was considered as the ratio between the normalised LC3-II protein levels in presence and absence of BafiA1. *n* = 3.

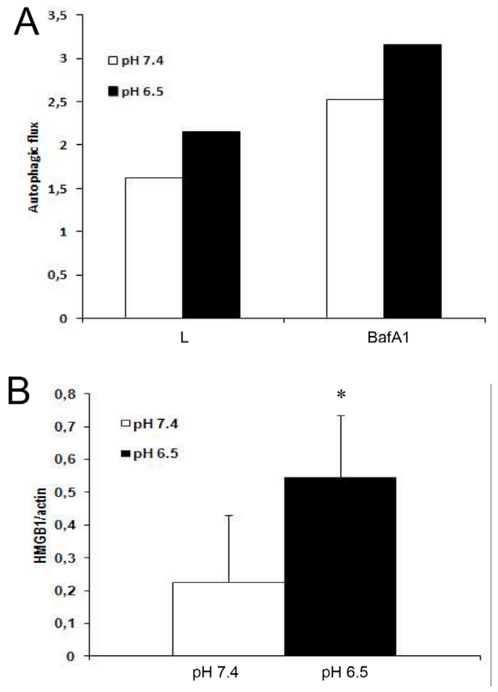


**Supplementary Figure S4: Densitometric quantification of western blot analysis shown in Figure 3C.** HOS cells cultured at pH 7.4 and 6.5, in presence of BafA1 (50 nM) for 2 h before collection, or of the lysosomal inhibitors (L, Pepstain A/E64d, 10 µg/mL), or with complete medium (M); A. Quantification of the ratio between the normalised LC3-II protein levels of cells treated or not treated with Baf1 or with L (autophagc flux). *n* = 1B. Quantification of HGMB1 in HOS cells (not treated, M) cultured at pH 7.4 and 6.5. *n* = 5, **p* = 0.0225.

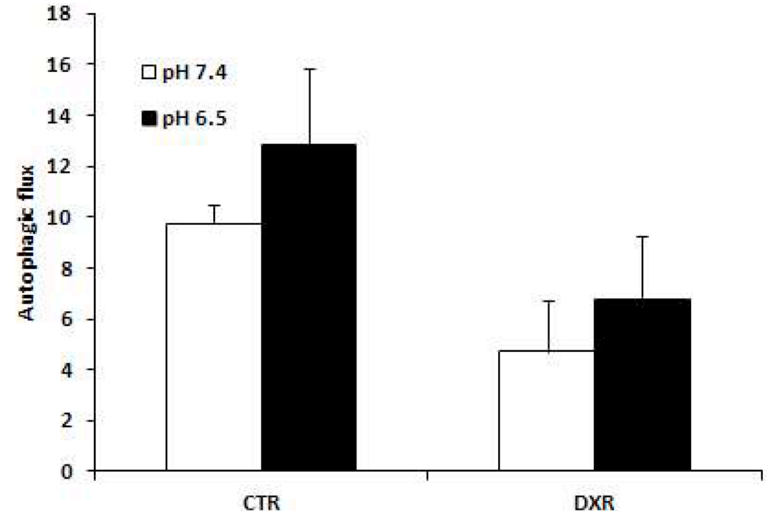


**Supplementary Figure S5: Densitometric quantification of western blot analysis shown in Figure 3D.** HOS cells treated for 24 h with DXR, in presence or absence of BafA1 at saturating concentration (autophagic flux) at different pH (7.4 and 6.5). *n* = 3.

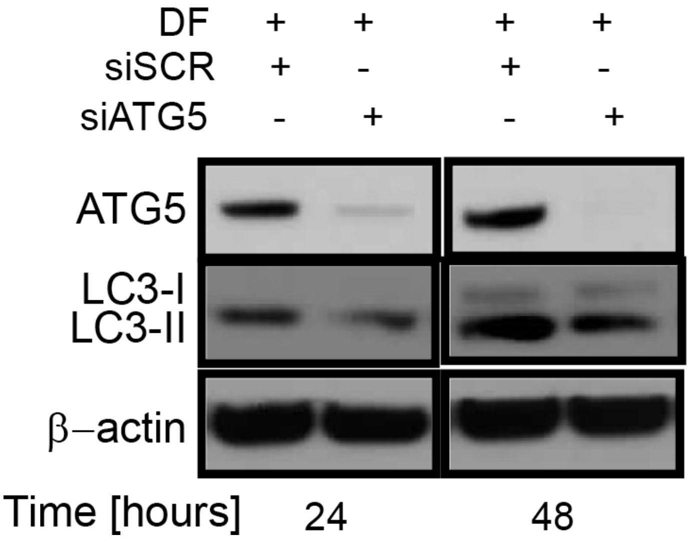


**Supplementary Figure S6: Western blot analysis of the ATG5 silencing in HOS cells.** The silencing efficently blocked the autophagic flux, as demonstrated by the reduction of ATG5 and LC3-II expression in the cells trasfected with siATG5 (siSCR, scrambled control).
